# Molecular characterisation of new organisation of *plnEF* and *plw* loci of bacteriocin genes harbour concomitantly in *Lactobacillus plantarum* I-UL4

**DOI:** 10.1186/s12934-015-0280-y

**Published:** 2015-06-16

**Authors:** Hui Fong Tai, Hooi Ling Foo, Raha Abdul Rahim, Teck Chewn Loh, Mohd. Puad Abdullah, Kimura Yoshinobu

**Affiliations:** Department of Bioprocess Technology, Faculty of Biotechnology and Biomolecular Sciences, Universiti Putra Malaysia, 43400 UPM Serdang, Selangor, Malaysia; Institute of Bioscience, Universiti Putra Malaysia, 43400 UPM Serdang, Selangor, Malaysia; Department of Cell and Molecular Biology, Faculty of Biotechnology and Biomolecular Sciences, Universiti Putra Malaysia, 43400 UPM Serdang, Selangor, Malaysia; Department of Animal Science, Faculty of Agriculture, Universiti Putra Malaysia, 43400 UPM Serdang, Selangor, Malaysia; Institute of Tropical Agriculture, Universiti Putra Malaysia, 43400 UPM Serdang, Selangor, Malaysia; Department of Biofunctional Chemistry, Graduate School of Environmental and Life Sciemce, Okayama University, Okayama, Japan

**Keywords:** Molecular characterisation, Genetic organisation, Genetic location, *pln* genes, *plnEF* locus, *plw* locus, Bacteriocin gene, *Lactobacillus plantarum* I-UL4

## Abstract

**Background:**

Bacteriocin-producing Lactic acid bacteria (LAB) have vast applications in human and animal health, as well as in food industry. The structural, immunity, regulatory, export and modification genes are required for effective bacteriocin biosynthesis. Variations in gene sequence, composition and organisation will affect the antimicrobial spectrum of bacteriocin greatly. *Lactobacillus plantarum* I-UL4 is a novel multiple bacteriocin producer that harbours both *plw* and *plnEF* structural genes simultaneous which has not been reported elsewhere. Therefore, molecular characterisation of bacteriocin genes that harboured in *L. plantarum* I-UL4 was conducted in this study.

**Results and discussion:**

Under optimised conditions, 8 genes (*brnQ1*, *napA1*, *plnL*, *plnD*, *plnEF*, *plnI*, *plnG* and *plnH*) of *plnEF* locus and 2 genes (*plw* and *plwG*) of *plw* locus were amplified successfully from genomic DNA extracted from *L. plantarum* I-UL4 using specific primers designed from 24 *pln* genes selected randomly from reported *plw*, *plS*, *pln423* and *plnEF* loci. DNA sequence analysis of the flanking region of the amplified genes revealed the presence of two *pln* loci, UL4-*plw* and UL4-*plnEF* loci, which were chromosomally encoded as shown by Southern hybridisation. UL4-*plw* locus that contained three ORFs were arranged in one operon and possessed remarkable amino acid sequence of LMG2379-*plw* locus, suggesting it was highly conserved. Interestingly, the UL4-*plnEF* locus appeared to be a composite *pln* locus of JDM1-*plnEF* and J51-*plnEF* locus in terms of genetic composition and organisation, whereby twenty complete and one partial open reading frames (ORFs) were aligned and organised successfully into five operons. Furthermore, a mutation was detected in *plnF* structural gene which has contributed to a longer bacteriocin peptide.

**Conclusions:**

Plantaricin EF and plantaricin W encoded by *plnEF* and *plnW* loci are classified as class I bacteriocin and class II bacteriocin molecules respectively. The concurrent presence of two *pln* loci encoding bacteriocins from two different classes has contributed greatly to the broad inhibitory spectrum of *L. plantarum* I-UL4. The new genetic composition and organisation of *plnEF* locus and concurrent presence of *plnEF* and *plnW* loci indicated that *L. plantarum* I-UL4 is a novel multiple bacteriocin producer that possesses vast potentials in various industries.

## Background

Lactic acid bacteria (LAB) is a group of bacteria frequently isolated from food. LAB genera that have important role in food and animal industries are *Lactococcus*, *Leuconostoc*, *Pediococcus*, *Lactobacillus*, and *Streptococcus* [1]. Extensive reports have shown LAB have capability to produce various compounds, such as acetic acid, hydrogen peroxide, ethanol, diacetyl and bacteriocins that contribute to the inhibitory effects to pathogenic microorganisms [2, 3]. Bacteriocins are ribosomal synthesised peptides or proteins that release extracellularly to inhibit bacteria closely related to the producing strains [4]. The inhibitory activities are mainly mediated through pore formation on cytoplasmic membrane or by inhibiting cell wall synthesis of sensitive bacteria [5–7]. Bacteriocins and bacteriocin-producing LAB have received special attention due to their potential applications in human and animal health, as well as in food industry [8–11]. The structural, immunity, regulatory, export and modification genes of bacteriocin that commonly arrange into one or more operon structures are required for effective bacteriocin biosynthesis [12, 13].

Despite a number of bacteriocins produced by *Lactobacillus plantarum* that generally known as plantaricin have been described [14–18], only a few plantaricin (*pln*; with italic formatted is used to describe gene) loci have been characterised genetically. The structure and organisation of *pln* loci may be simple or complex. The relatively simple *pln* loci are found in one operon, such as *plw* locus that encodes Class I two-peptide plantaricin W [19], *plS* locus that encodes Class IIb plantaricin S [20] and *pln423* locus that encodes Class IIa plantaricin 423 [21]. The relatively complex *pln* locus is *plnEF* locus that distributes widely among *L. plantarum* isolated from various ecological niches. The well characterised *plnEF* locus has been reported for *L. plantarum* C11 [22], WCFS1 [23], JDM1 [24], J23 [25], J51 [26], NC8 [27] and V90 [28]. The reported *plnEF* loci have been designated as *plnEF* locus for *L. plantarum* JDM1, C11, WCFS1, V90, J51, NC8 and J23 respectively. The size of the reported *plnEF* loci are between 18 and 19 kb with 22 to 26 genes and they are organised in five to six operons in mosaic like structure encoding four types of class IIb plantaricins and three regulatory networks [28].

Probiotic effects of bacteriocin-containing postbiotic produced by *L. plantarum* have been reported for rats and livestock animals [29–34]. The bacteriocin-containing postbiotic of *L. plantarum* I-UL4 isolated from *tapai ubi* (fermented tapioca, a Malaysian traditional fermented food) has been shown to have broad inhibitory spectrum against various Gram-positive (*Bacillus cereus*, *Staphylococcus aureus*, *Streptococcus pneumoniae*, *Enterococcus faecalis*, *Enterococcus faecium* and *Pediococcus acidilactici*) and Gram-negative bacteria (*Escherichia coli* and *Salmonella typhimurium*) [35, 36]. According to Moghadam et al. [37]*, L. plantarum* I-UL4 is a multiple bacteriocin producer that harbours both *plw* and *plnEF* structural genes. The simultaneous detection of both *plw* and *plnEF* that encode for plantaricin W and plantaricin EF respectively has not been reported elsewhere [37]. Furthermore, the genetic loci of *plnEF* are in high plasticity and possess many variable regions with respect to their mosaic genetic composition and regulatory network [28]. Hence, the characterisation of *pln* loci is important as variations in gene sequence, gene composition and organisation will affect the antimicrobial spectrum of bacteriocin that release in extracellular environment. In addition, new open reading frame (ORF) can be discovered in close proximity to the known bacteriocin genes. Therefore, molecular characterisation of *plnEF* and *plw* loci of bacteriocin genes that harbour concomitantly in *Lactobacillus plantarum* I-UL4 were conducted in this study.

## Results and discussion

### *pln* genes of *L. plantarum* I-UL4

The *pln* genes of *L. plantarum* I-UL4 were detected by PCR using gene-specific primers designed from 24 *pln* genes selected randomly from reported *plw* [19], *plS* [20], *pln423* [21] and *plnEF* [22, 27] loci. Under optimised conditions, 8 genes (*brnQ1*, *napA1*, *plnL*, *plnD*, *plnEF*, *plnI*, *plnG* and *plnH*) of *plnEF* locus and 2 genes (*plw* and *plwG*) of *plw* locus were amplified successfully. The identities of amplified *pln* genes were further confirmed by DNA sequence analyses, whereby high DNA sequence identity (ranging from 96 to 100%) that correspond to respective *pln* gene was obtained for all amplified DNA fragments (Table 1). In contrast, 11 *pln* genes (*plnA*, *plnB*, *plnC*, *plnM*, *plnN*, *plnO*, *plnP*, *plnJ*, *plnK*, *plNC8*, *plNC8HK*) of *plnEF* loci and all the selected genes from *plS* and *pln423* loci were absent in the studied strain as confirmed further by gradient PCR analysis, inferring that *L. plantarum* I-UL4 harbours *plw* and *plnEF* loci simultaneously as reported by Moghadam et al. [37]. Although several studies reported the presence of *plnEF* gene in bacteriocinogenic *L. plantarum* isolated from fermented foods by PCR screening, none of the reported isolates harboured *plw* structural gene [38–40] simultaneously. In addition, only *plnEF* structural gene was found in the complete genome sequence of *L. plantarum* WCFS1 [23] and *L. plantarum* JDM1 [24]. Therefore, *L. plantarum* I-UL4 is the first *L. plantarum* strain that has been reported to harbour both *plw* and *plnEF* structural genes concomitantly, which have contributed greatly to the broad inhibitory spectrum of bacteriocin-containing postbiotic produced by *L. plantarum I*-UL4 against various Gram-positive (*Bacillus cereus, Staphylococcus aureus, Streptococcus pneumoniae, Enterococcus faecalis, Enterococcus faecium* and *Pediococcus acidilactici*) and Gram-negative bacteria (*Escherichia coli and Salmonella typhimurium*) as reported by Lim [35] and Thanh et al. [36]. Moreover, the *pln* genes in *plnEF* locus of *L. plantarum* I-UL4 [UL4-*plnEF* locus; for simplicity, the ORF, peptide or locus of a strain was abbreviated as (name of the strain)-(*ORF*, peptide or locus)] were different from the reported *plnEF* loci.

**Table 1:** Nucleotide sequence characteristics of PCR-amplified *pln* genes harboured in *Lactobacillus plantarum* I-UL4 in comparison to the *pln* genes reported for *Lactobacillus plantarum* JDM1, C11, WCFS1, V90, J51, NC8, J23 and LMG2379

*pln* genes	Length (bp)	Function of gene	Nucleotide sequence identity (%)							
			JDM1	C11	WCFS1	V90	J51	NC8	J23	LMG2379
*brnQ1*	1,088	Amino acid transport protein	98	ND	98	ND	98	ND	99	ND
*napA1*	738	Na^+^/H^+^ antiporter	98	ND	99	ND	99	99	98	ND
*plnL*	382	Putative immunity protein	96	96	96	96	96	96	96	ND
*plnD*	365	Response regulator	100	96	96	95	96	100	96	ND
*plnI*	558	Immunity	98	98	98	98	98	98	99	ND
*plnEF*	369	Prebacteriocin	98	99	99	98	99	99	99	ND
*plnG*	394	ABC transporter	99	99	99	98	99	ND	99	ND
*plnH*	926	Accessory protein	99	98	98	98	98	ND	98	ND
*plw*	279	Prebacteriocin	ND	ND	ND	ND	ND	ND	ND	100
*plwG*	975	ABC transporter	ND	ND	ND	ND	ND	ND	ND	99

*ND* not detected, the respective gene was not detected in the reference strain.

### Characterisation of UL4-*plw* locus

The upstream and downstream DNA sequences of *plw* and *plwG* were amplified and analysed from genomic DNA of *L. plantarum* I-UL4 (*plw* loci of *L. plantarum* I-UL4 were deposited at GenBank/EMBL/DDBJ with accession number of GU322921). A contig of 2.77 kb termed UL4-*plw* locus was successfully assembled and DNA sequence analysis of UL4-*plw* locus revealed the presence of three ORFs (*plwβ*, *plwα* and *plwG*) that arranged in one operon with same orientation. Both *plwβ* and *plwα* were 100% identical to LMG2379-*plwβ* and LMG2379-*plwα* respectively [19]. *plwα* and *plwβ* are the structural genes that encode for Class I two-peptide lantibiotic, plantaricin W, whereby the mature peptides are modified to contain lanthionine, methyl Lanthionine and dehydrated residues [19]. The last ORF, *plwG*, that encoded for ABC-transporter was highly similar (more than 99.7% identities) to LMG2379-*plwG* [19].

### Characterisation of UL4-*plnEF* locus

**Figure 1:**
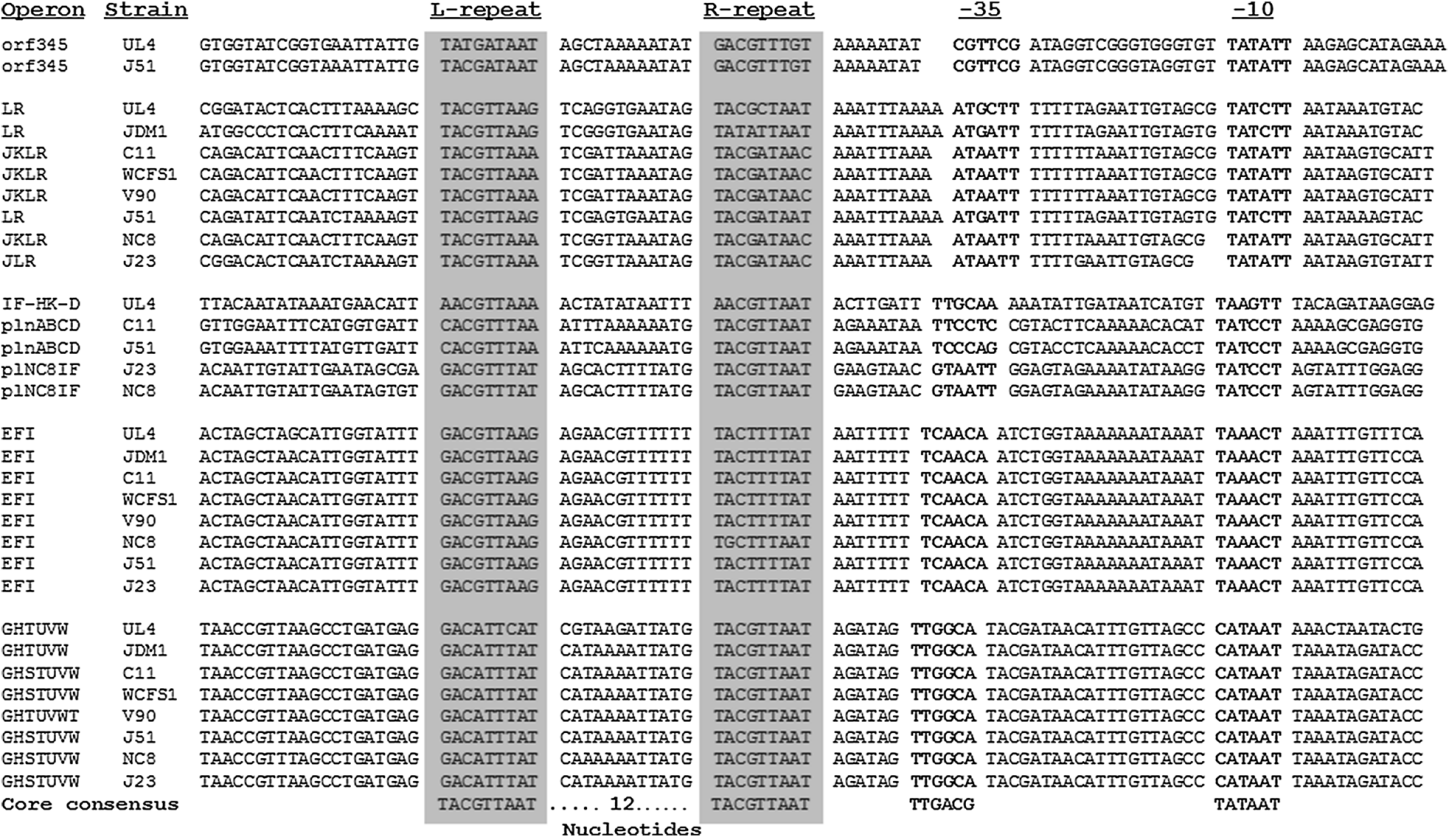
Putative promoters of UL4-*plnEF* locus that searched by DNA sequence alignment and comparison to the promoter sequences reported for *plnEF* loci. The promoters that identified in UL4-*plnEF* locus were consisted of a pair of direct repeat which was located at the upstream of −35 region. Each pair of the repeats was separated by a spacer of 12 nucleotides that are highlighted in *grey-boxes*. Putative −35 and −10 sequences are indicated with *boldface.*

The upstream and downstream DNA sequences of *brnQ1*, *napA1*, *plnL*, *plnD*, *plnEF*, *plnI*, *plnG* and *plnH* in *plnEF* locus were successfully amplified and sequenced from genomic DNA of *L. plantarum* I-UL4 and a contig of 17.58 kb that designated as UL4-*plnEF* locus was obtained by careful alignment and assembly (*plnEF* loci of *L. plantarum* I-UL4 were deposited at GenBank/EMBL/DDBJ with accession number of GU138149). The amino acid sequence of deduced peptides encoded by UL4-*plnEF* locus and the reported *plnEF* loci are shown in Table 2. Figure 1 shows the putative promoters that were searched manually by sequence alignment and comparison to the promoter sequences reported for *pln* operons. The promoter sequences that identified in the UL4-*plnEF* locus were consisted of a pair of direct repeat which was located at the upstream of −35 region. Each pair of the repeats was separated by a spacer of 12 nucleotides that rich in adenine and thymine. The characteristic of promoters identified in UL4-*plnEF* locus were highly identical to the reported *plnEF* loci [28]. The direct repeat pair is important for the regulation of bacteriocin biosynthesis at transcriptional level as this consensus direct repeat serves as DNA binding sites for response regulator (RR) to initiate the transcription process [41, 42]. Changes in nucleotide sequence of the repeat such as point substitutions, deletion of repeat or alteration in the length of spacer region can abolish or reduce the binding of RR and subsequently suppress the gene expression [43]. The promoter motifs of *pln* operons were also found in other bacteriocin systems such as gene cluster of sakacin A [44, 45], sakacin P [46, 47], carnobacteriocin A [48], carnobacteriocin B2 [49] and enterocin A [50, 51], indicating similar regulatory mechanism was used for the production of various bacteriocins.

**Table 2:** Characteristics of the predicted ORFs encoded by UL4-*plnEF* locus amplified from *Lactobacillus plantarum* I-UL4

Predicted ORFs	Orientation (+ or −)	Nucleotide coordinates	Gene and peptide length (bp: aa)	15 bp upstream of the start codon (5′–3′)	Homologous gene and function	Re-designated as
*ef1*	+	829–2,205	1,377: 458	GGAGGAGAGACGACT	*brnQ1*: amino acid transporter	*brnQ1*
*ef2*	+	2,238–3,434	1,197: 398	TAAGACTTTTGATGG	*napA1*: Na^+^/H^+^ antiporter	*napA1*
*ef3*	+	3,809–3,982	174: 57	GAAAAGGTGATTAAA	*orf3*: putative prebacteriocin	*orf3*
*ef4*	+	3,998–4,177	180: 59	AAAGAAGTGGTAAAA	*orf4*: putative prebacteriocin	*orf4*
*ef5*	+	4,283–4,525	243: 80	TTGTTTGTTCTTTTA	*orf5*: putative immunity protein	*orf5*
*ef6*	–	4,970–5,083	114:37	GTAAGGCACACGTTA	*plnR*: unknown	*plnR*
*ef7*	–	5,108–5,776	669: 222	CTCGGGGGATTATAA	*plnL*: putative immunity protein	*plnL*
*ef8*	+	6,369–6,536	168: 55	GGAGGGGTTATTATT	Putative induction factor	*UL4IF*
*ef9*	+	6,554–7,894	1,341: 446	TAGGTGGTGTTCCAC	*HK*: histidine Protein Kinase	*UL4HK*
*ef10*	+	7,895–8,638	744: 247	TTGGAGGAAGAATGA	*plnD*: response regulator	*plnD*
*ef11*	–	8,932–9,705	774: 257	GGGGGAATTTTAACT	*plnI*: immunity protein	*plnI*
*ef12*	–	9,784–9,963	180: 59	GGGAGATCAACAATT	*plnF*: plantaricin EF precursor	*plnF*
*ef13*	–	9,988–10,158	171: 56	CAAGGGGGATTATTT	*plnE*: plantaricin EF precursor	*plnE*
*ef14*	+	10,424–12,574	2,151: 716	GAGGGGAGTACAAGT	*plnG*: ABC transporter	*plnG*
*ef15*	+	12,591–13,967	1,377: 458	GGGGGAAACTGAATA	*plnH*: accessory protein	*plnH*
*ef16*	+	14,057–14,746	690: 229	CGAAAGAGGTAAGTA	*plnT*: unknown	*plnT*
*ef17*	+	14,814–15,482	669: 222	CTTGGGAGGCTTGGT	*plnU*: unknown	*plnU*
*ef18*	+	15,569–16,249	681: 226	TGGATGTGAAGGAGC	*plnV*: unknown	*plnV*
*ef19*	+	16,343–17,029	687: 228	GATGGAGTGGATGAA	*plnW:* unknown	*plnW*
*ef20*	+	17,167–17,370	204: 67	AGGAGTTTGGTAAGT	*orfZ1*: unknown	*UL4orfZ1*
*ef21*	–	17,465–>17,588	>124:>40	ND	*DHelicase*: DNA helicase	*DHelicase*

Underlined nucleotides are putative RBS. No RBS could be detected for *ef5* which was re-designated as *plnR*. *ef8* did not show homology to any entries in database but the deduced peptide sequence contained GG motif. *ef21* was partially sequenced and hence upstream sequence of *ef21* is not available.

*ND* not detected.

**Figure 2:**
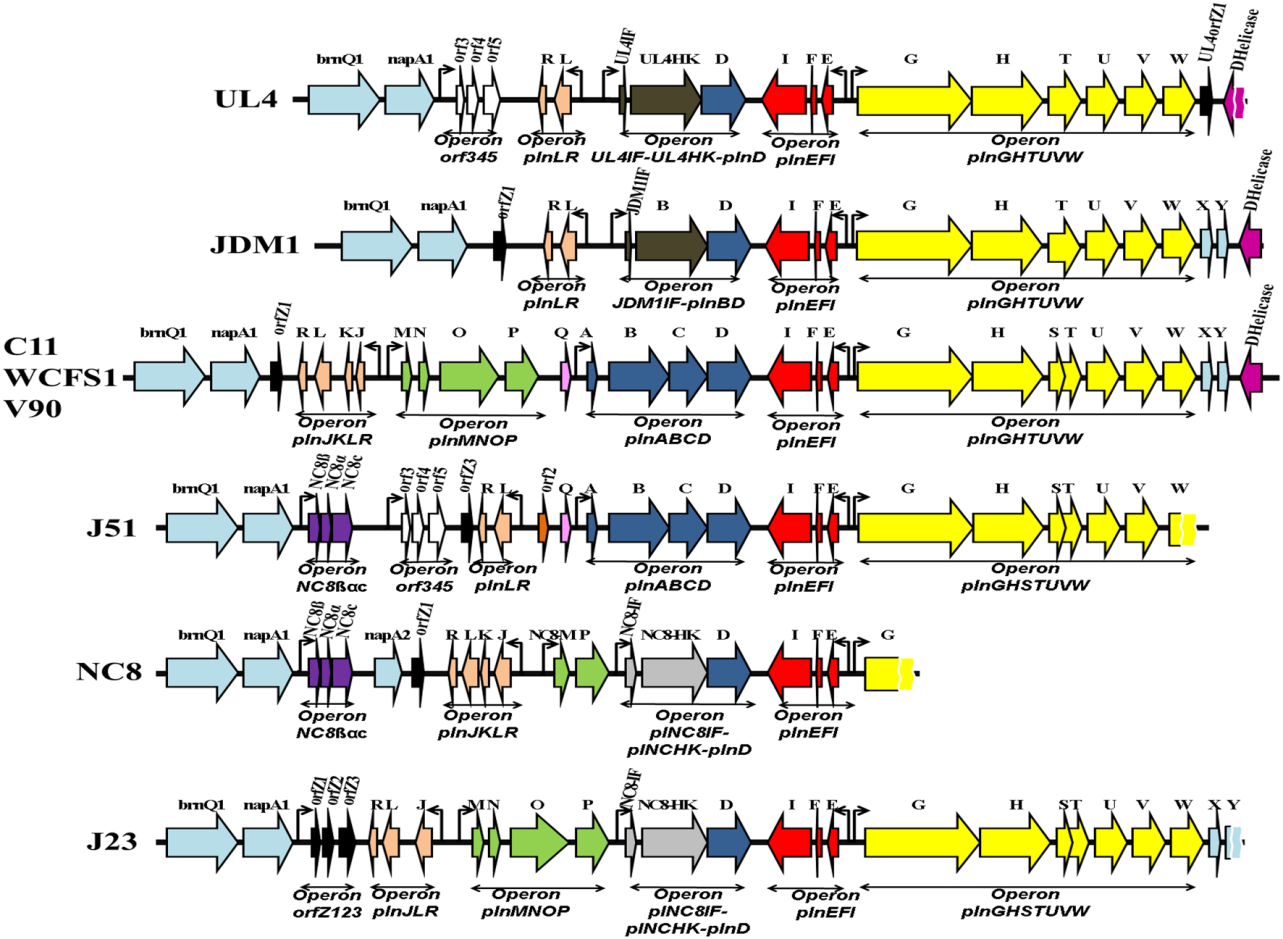
Genetic map for the comparison of genetic organisation of UL4-*plnEF* locus and reported *plnEF* loci of *L. plantarum* strains. ORFs are represented by *arrow-blocks*. The promoter sequences are indicated by small *black arrows*. *brnQ1* and *napA1* signify the upper boundary while *DNA helicase* signify the lower boundary of *plnEF* loci. The DNA sequence of UL4-*DHelicase*, J51-*plnW*, NC8-*plnG* and J23-*plnY* was partially analysed. C11-, WCFS1- and V90-*plnEF* loci were identical. However, *brnQ1*, *napA1*, *plnX*, *plnY* and *DHelicase* were not described in C11; *brnQ1, napA1* and *DHelicase* were not described in V90; *plnS* and *plnT* were “fused” in V90. The genetic map was generated using information retrieved from GenBank with accession number of CP001617 (JDM1), X94434 (C11), NC_004567 (WCFS1), FJ809773 (V90), DQ340868 (J51), AF522077 (NC8) and DQ323671 (J23) respectively.

Biocomputational analyses of UL4-*plnEF* locus revealed the presence of 20 complete and one partial ORFs. The comparison of genetic organisation of UL4-*plnEF* locus and reported *plnEF* loci are illustrated in a genetic map as shown in Figure 2. Five putative operons (*orf345, plnLR, UL4IF*-*UL4HK*-*plnD, plnEFI* and *plnGHTUVW*) that preceded by a putative promoter were deduced from the UL4-*plnEF* locus. The operons of *orf345*, *plnLR* and *plnEFI* were predicted to encode for a two-peptide bacteriocin and immunity protein respectively. The *UL4IF*-*UL4HK*-*plnD* operon was predicted to regulate bacteriocin production at transcriptional level. The last predicted operon, *plnGHTUVW,* was responsible for maturation and secretion of bacteriocins and bacteriocin-like peptides as proposed by Diep et al. [22, 28]. Three ORFs of *brnQ1*, *napA1* and *DHelicase* that amplified and sequenced from the genomic DNA of *L. plantarum* I-UL4 were also found in the reported operons. However, their functions have not been related to any bacteriocin production.

**Figure 3:**
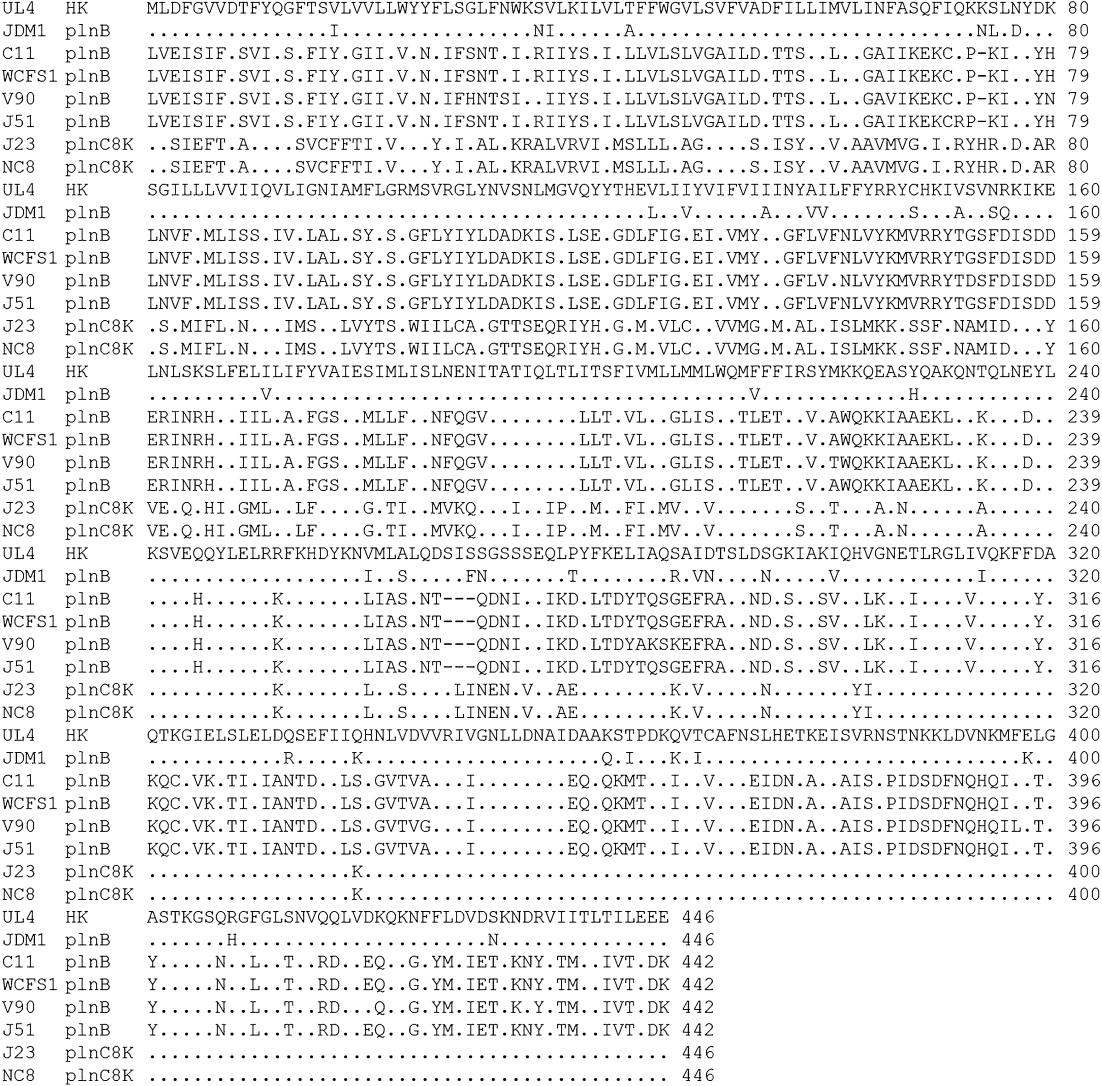
DNA sequence alignment of UL4HK with HPK of reported *plnEF* loci. Low amino acid sequence identity at N-terminal receptor domain of HPKs was detected. Amino acids that identical to UL4HK are represented by *dot*.

Three-component regulatory system of *UL4IF*-*UL4HK*-*plnD* was detected in the UL4-*plnEF* locus as compared to *plnABCD* or *plNC8IF*-*plNCHK*-*plnD* regulatory operon that reported for *plnEF* locus by Diep et al. [28]. *UL4IF* was found to encode a putative induction factor (IF) that usually activates transcription process of regulated genes. The leader peptide of UL4IF contained a double-glycine (GG) motif and the mature peptide consisted of 28 amino acids. The calculated pI and the MW of the mature peptide was 11.26 and 3321.98 Da, respectively. The IFs that identified in bacteriocin systems are a small bacteriocin-like peptide having several physicochemical properties of bacteriocin. Both IF and bacteriocin are synthesised as a precursor peptide containing GG leader peptide and hence the same maturation and secretion system has been suggested for both IF and bacteriocin. In addition, the mature peptide of both IF and bacteriocin has high pI and low MW. Although IF and bacteriocin share several physicochemical properties, IF can be discriminated from bacteriocin in the way that IF possess little or no bacteriocin activity and the gene encoding IF is always located in the same transcription unit and preceded the gene encoding histidine protein kinase (HPK) and RR [47, 48, 50, 52, 53]. *UL4HK* that encoded for HPK and *plnD* that encoded for RR were located at downstream and in the same transcriptional unit of *UL4IF*. DNA sequence alignment of UL4HK with HPK of reported *plnEF* loci revealed low amino acid sequence identities at N-terminal receptor domain of HPKs (Figure 3). On the contrary, the C-terminal domain of HPKs shared significant nucleotide and amino acid sequence identity. Nevertheless, the regulatory operons of the reported *plnEF* loci were semi-conserved in which *plnD* was found in all regulatory operons regardless of the HPK type, suggesting that the interaction between IF and HPK is highly specific while the interaction between HPK and RR is less specific. The results obtained in this study were further supported by the notion of antimicrobial activity of *L. plantarum* J23 containing *plNC8IF*-*plNCHK*-*plnD* regulatory operon that only could be detected when induced by plNC8IF and not plnA [25].

**Figure 4:**
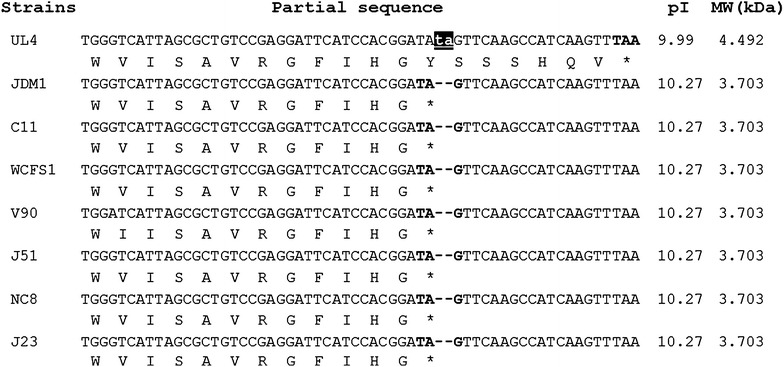
Nucleotide and deduced amino acid sequences of plnF peptide (partial sequence). Stop codon is indicated by *boldface* and *asterisk*. The nucleotide insertions are shown as *small cap* and highlighted in *black* background. The pI and MW are the calculated pI and MW of the corresponding mature peptides.

The *plnEFI* operon encoded for plantaricin EF and its cognate immunity protein was present in *L. plantarum* I-UL4. No variation of amino acid was detected in UL4-plnE as compared to the reported plnE. However, UL4-*plnF* was seven amino acids longer than the reported plnF peptide due to the insertion of two nucleotides at the stop codon resulting in additional translation of seven amino acids N′- YSSSHQV- C′ prior to TAA stop codon (Figure 4). Thus, the mature UL4-plnF contains 41 amino acids with the calculated MW of 4.492 kDa, as compared to 34 amino acids of the reported plnF. The pI of UL4-plnF is 9.99 which is 0.28 unit lower than the plnF of the reported *plnEF* loci. A similar case was demonstrated by Rojo-Bezares et al. [25] for J23-plnJ, whereby J23-plnJ was reported to be 28 amino acids longer than the reported plnJ (55 amino acids) and a great reduction in antimicrobial activity was observed in the J23-plnJ peptide. However, the antimicrobial activity of the UL4-plnF has yet to be determined.

Another bacteriocin-like operon *orf345* that previously described in *L. plantarum* J51 [26] was detected in UL4-*plnEF* locus as well. Operon UL4-*orf345* contained three ORFs of *orf3, orf4* and *orf5*, which was highly identical to those described for *L. plantarum* J51. However, one amino acid mismatch was detected in both orf3 and orf4 [26] respectively and GG leader peptide was detected in both UL4-orf3 and UL4-orf4 [54]. The mature peptide of UL4-orf3 and UL4-orf4 has highly cationic property with calculated pI of 11.45 and 9.87 respectively. Hence, UL4-*orf345* operon resembles a bacteriocin and immunity operon encodes for a two-peptide bacteriocin together with its cognate immunity protein.

A class II bacteriocin, plantaricin JK together with its dedicated immunity and a hypothetical protein with unknown function were encoded by *plnJKLR* operon [22]. The *plnJKLR* operon was found as a degenerated operon, *plnLR*, in the UL4-*plnEF* locus. In addition, similar degenerated form of *plnJKLR* operon was reported commonly in *plnEF* loci in the form of *plnJLR* or *plnLR* operon [28].

UL4-*orfZ1* showed high nucleotide sequence identity to *orfZ1* of *L. plantarum* JDM1, C11, WCFS1, V90, NC8 and J23. The *orfZ1* is the member of putative bacteriocin-like operon, namely *orfZ123* operon, which consists of three ORFs. The *orfZ2* was reported to encode a peptide with GG motif leader peptide, while the *orfZ1* and the *orfZ3* were encoded for peptides with unknown functions [25]. However, the degenerated form of *orfZ123* operon (*orfZ1* alone) was detected in *L. plantarum* I-UL4. According to Diep et al. [28], this operon was degenerated greatly, whereby either *orfZ1* or *orfZ3* was detected in the reported *plnEF* loci.

Bacteriocins with GG leader peptides were processed and secreted by a dedicated ABC-transporter. A highly conserved secretion operon, either *plnGHTUVW* or *plnGHSTUVW* was found in those reported *plnEF* loci. The major difference between *plnGHTUVW* or *plnGHSTUVW* operon is that *plnT* of *plnGHTUVW* operon is a fusion gene of *plnS* and *plnT* of *plnGHSTUVW* operon [28]. The secretion operon that detected in UL4-*plnEF* locus was *plnGHTUVW* operon. UL4-*plnG* and UL4-*plnH* encoded for a hybrid ABC-transporter and its corresponding accessory protein, respectively. This hybrid ABC-transporter consists of a N-terminal proteolytic, a core trans-membrane spanning and a C-terminal ATP-binding domain. UL4-*plnT* appeared to be a fusion gene of *plnS* and *plnT* found in C11, WCFS1, J51, NC8 and J23. UL4-plnT shared 99.1 and 96.9% amino acid sequence identity to JDM1- and V90-plnT, respectively. The *plnTUVW* encoded putative proteins that belong to Abi family and they contained protease CAAX motif [55]. It was noted that some identified bacteriocin immunity proteins belong to Abi family and Kjos et al. [56] have shown the involvement of several Abi proteins in bacteriocin self-immunity [28, 57, 58]. However, the role of *plnTUVW* in bacteriocin system still remains unknown.

### Genetic location of *plw* and *plnEF* loci

**Figure 5:**
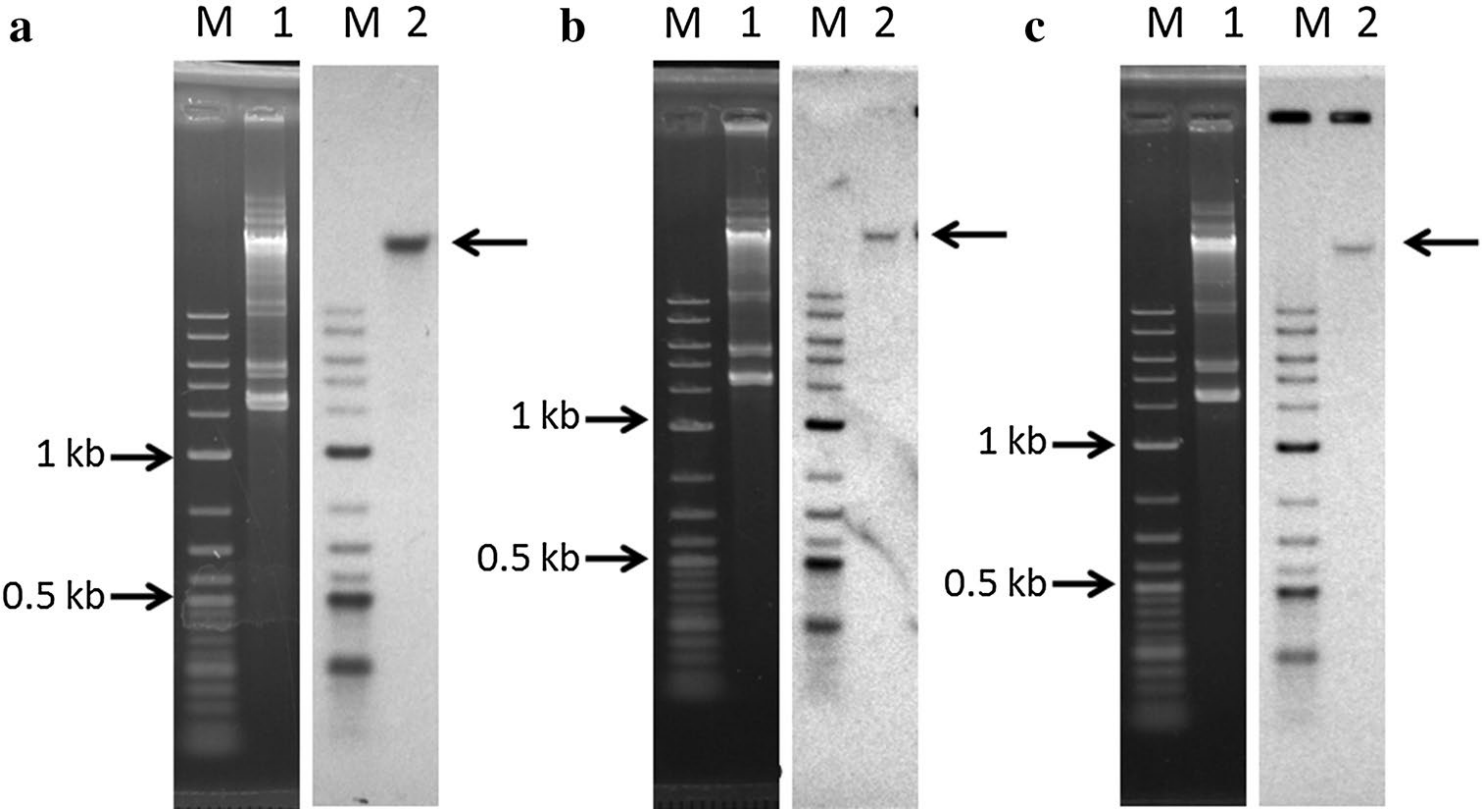
Southern blot analyses of genetic location of *plnEF* and *plw* loci of *Lactobacillus plantarum* I-UL4. *Left panel* agarose gel electrophoresis of genomic DNA. *Right panel* Southern blot analyses for the hybridisation of genomic DNA with the targeted 16S rDNA (**a**), *plnEF* (**b**) or *plw* (**c**). *Lane M* Biotinylated 2-Log DNA Ladder; *Lane 1* genomic DNA of *L. plantarum* I-UL4; *Lane 2* Hybridisation signals of chromosomal DNA and the targeted 16S rDNA (**a**), *plnEF* (**b**) or *plw* (**c**) as shown by the *thick arrows*.

The genetic location of *plw* locus has not been reported elsewhere. However, *plnEF* locus of *L. plantarum* WCFS1 [23] and *L. plantarum* JDM1 [24] have been reported to be located on chromosomal DNA. *L. plantarum* I-UL4 that employed in this study harboured multiple plasmids as shown by agarose gel electrophoresis of the genomic DNA (Figure 5). Therefore, Southern hybridisation of genomic DNA with three DNA probes, namely 16S_probe_, EF_probe_ and W_probe_, were carried out to determine the genetic location of UL4-*plnEF* and UL4-*plw* loci that harboured in *L. plantarum* I-UL4. The 16S_probe_ generated in this study was 100% complementary to the 16S rDNA sequence of *L. plantarum* I-UL4, which was specific to chromosomal DNA rather than plasmid DNA. The hybridisation signals generated by 16S_probe_ would differentiate and confirm the identification of chromosomal DNA band from plasmid DNA bands separated by agarose gel electrophoresis. The hybridisation signal of EF_probe_ and W_probe_ were detected at the same DNA band as the 16S_probe_ (Figure 5), indicating that *plnEF* and *plw* loci were located on chromosomal DNA since the location of 16S rDNA is only found in chromosomal DNA of *L. plantarum* I-UL4.

## Conclusions

*L. plantarum* I-UL4 was shown to be a multiple bacteriocin producer, harbouring *plw* and new mosaic *plnEF* loci that chromosomally encoded as shown by Southern hybridisation. This is the first report of a *L. plantarum* strain harbouring the combination of *plw* and *plnEF* loci concomitantly. The plantaricin W and plantaricin EF encoded by *plw* and *plnEF* loci respectively are two different classes of bacteriocin, in which plantaricin W is a class I bacteriocin molecule while plantaricin EF is a class II bacteriocin molecule. UL4-*plw* locus was highly conserved and contained remarkable amino acid sequence of LMG2379-*plw* locus. However, the UL4-*plnEF* locus appeared to be a composite *pln* locus of JDM1-*plnEF* and J51-*plnEF* locus in terms of genetic composition and organisation. The new genetic composition and organisation of *plnEF* locus and concurrent presence of *plnEF* and *plnW* loci indicated that *L. plantarum* I-UL4 is a novel multiple bacteriocin producer that possesses vast potentials in various industries.

## Methods

### Bacterial strain and culture conditions

*L. plantarum* I-UL4 isolated from fermented tapioca, “*tapai ubi*” was used in this study [35]. The strain was deposited at the Microbial Culture Collection Unit (UNICC) of Institute of Bioscience, Universiti Putra Malaysia with deposition number UPMC5. The studied strain was grown in de Man-Rogosa-Sharpe (MRS) media (Merck, Germany) at 30°C [59] under anaerobic condition.

### Genomic DNA extraction

The genomic DNA of *L. plantarum* I-UL4 was extracted using the method described by de los Reyes-Gavilán et al. [60] with minor modifications. Briefly, a single colony of *L. plantarum* 1-UL4 was inoculated into 10 ml of MRS broth and incubated at 30°C for overnight. Bacteria cells were then harvested from 1 ml of overnight culture by centrifugation at 16,000×*g* for 10 min at 4°C, followed by incubating the cell pellet for 1 h at 37°C in 200 μl of TEG buffer (25 mM Tris–HCl, 10 mM EDTA and 50 mM glucose at pH 8.0) containing 15 mg ml^−1^ lysozyme (Sigma, USA). Subsequently, 100 μl of 15% (w/v) SDS was added and mixed by gentle inversion to lyse the cells. Then, 300 μl of 3 M cold sodium acetate buffer (pH 5.2) was added and the mixture was inverted gently, followed by incubation on ice for 5 min. The mixture was then centrifuged at 16,000×*g* for 10 min at 4°C to precipitate the proteins. The resulting supernatant was transferred into a clean microcentrifuge tube and mixed with an equal volume of phenol:chloroform:isoamylalcohol solution (Amresco, USA). After centrifugation at 16,000×*g* for 15 min at 4°C, the aqueous phase containing DNA was transferred to a new microcentrifuge tube. Two sample volumes of cold absolute ethanol was then added to the aqueous phase, followed by gently mixing and incubated overnight at −20°C to precipitate the DNA. The mixture was centrifuged at 16,000×*g* for 15 min at 4°C to collect the DNA after overnight incubation. DNA pellet was then washed with 1 ml of 70% (v/v) cold ethanol and air-dried in a laminar air flow before re-suspended in 40 μl of 1× TE buffer (10 mM Tris–HCl and 1 mM EDTA at pH 7.0). RNA was removed by adding RNase A (Fermentas, Germany) to a final concentration of 0.4 mg ml^−1^, followed by incubation at 37°C for 15 min.

### Detection of *pln* genes

Gene-specific primers were designed specifically based on the published *pln* genes sequences selected randomly from *plw* [19], *plS* [20], *pln423* [21] and *plnEF* [22, 27] loci using internet-based software, PRIMER3 [61]. The specificity of each primer is listed in Table 3. PCR amplification was carried out in 25 μl reaction mixture containing 1× *Taq* buffer, 0.2 μM of each dNTPs, 2 mM MgCl_2_ (Fermentas, Germany), 0.08 μM of each forward and reverse primers, 1 unit of *Taq* DNA polymerase and 500 ng of genomic DNA extracted from the studied strain. PCR reaction was performed with MyCycler™ Thermal Cycler (BioRad, USA) using following program: (a) initial denaturation at 95°C for 5 min, (b) 30 cycles of denaturation at 95°C for 1 min, (c) annealing at 53°C for 1 min, (d) extension at 72°C for 1 min, and (d) final extension at 72°C for 7 min. PCR products were analysed using 1% (w/v) agarose gel electrophoresis. Gradient PCR with annealing temperature of 50–60°C was carried out for primers that produced negative results. Two positive controls (PLANT1 and LOWLAC primers that specific to partial 16S rDNA of *L. plantarum* [62]) and a negative control (without DNA template) were included in PCR amplification to monitor the functionality of DNA template, PCR components and contamination. The positive controls produced specific PCR fragment of 996 bp.

**Table 3:** PCR primers that designed for the detection of *pln* genes haboured in *Lactobacillus plantarum* I-UL4

Target gene	Function	Primer sequence (5′–3′)	Size (bp)	References
*brnQ1*	Amino acid transport protein	F: ATGCTCTTTGGGATGTTTTT	1,068	[23]
		R: ACGATGAAATAGCGGTGAGG		
*napA1*	Na-^+^/H^+^ antiporter	F: AAGTATTTACGCCCTGCCATTA	798	[23]
		R: TTAAACCCACACTGACGAAGAA		
*plnJ*	Prebacteriocin	F: TAACGACGGATTGCTCTG	475	[22]
		R: AATCAAGGAATTATCACATTAGTC		
*plnK*	Prebacteriocin	F: CTGTAAGCATTGCTAACCAATC	246	[22]
		R: ACTGCTGACGCTGAAAAG		
*plnL*	Putative immunity protein	F: TAGATGCCGCTCCGTAAAGT	442	[22]
		R: CGTTACCCTCGCCAAAGTG		
*plnM*	Unknown function	F: TGCTTGAAAGAATTACAGGATT	171	[22]
		R: CAAACGCAACCATCAAAATA		
*plnN*	Prebacteriocin	F: ATTGCCGGGTTAGGTATCG	146	[22]
		R: CCTAAACCATGCCATGCAC		
*plnO*	Glycosyl transferase group 2 family	F: CGGAGACCCTTTATTATTTTG	580	[22]
		R: TCTTCGGACCCCTCTGATT		
*plnP*	Protease CAAX family	F: TCCGAAAAGTATGGACAAATGA	437	[22]
		R: AAAGTTCCCCAAAGCAGACC		
*plnA*	Induction factor	F: CAAATTAAAGGTATGAAGCAACT	113	[22]
		R: TTCTTTACCTGTTTAATTGCAG		
*plnB*	Histidine kinase	F: CTGGCTTGTCGGAGTATGGT	531	[22]
		R: CGTCATTTAGGCTTGCTCTG		
*plnC*	Response regulator	F: GGCGACAGGAGATTTACAAGA	437	[22]
		R: CCACTTTATTTTTGGCAGTCAG		
*plnD*	Response regulator	F: TGAGGACAAACAGACTGGAC	415	[22]
		R: GCATCGGAAAAATTGCGGATAC		
*plnEF*	Prebacteriocin	F: GGCATAGTTAAAATTCCCCCC	428	[22]
		R: CAGGTTGCCGCAAAAAAAG		
*plnI*	Immunity	F: CGTTAATGGGTGATTGAGTTG	424	[22]
		R: AGTCTGCCTTTGAGCCTAGC		
*plnG*	ABC transporter	F : TGCGGTTATCAGTATGTCAAAG	454	[22]
		R: CCTCGAAACAATTTCCCCC		
*plnH*	Accessory protein	F: AGTTTTACGGGATTCGGTTT	986	[22]
		R: CTTTGCACCACGGTAATTGT		
*plw*	Prebacteriocin	F: AGTCGTCGTAAGAATGCTATTG	389	[19]
		R: TCACACGAAATATTCCA		
*plwG*	ABC transporter	F: GGTGTACTGGACTTAGGCATGG	1,034	[19]
		R: CGCTCTCGCAATCGTTATTC		
*plnC8*	Prebacteriocin	F: GGTCTGCGTATAAGCATCGC	207	[27]
		R: AAATTGAACATATGGGTGCTTTAAATTCC		
*plNC8HK*	Histidine kinase	F: AGCGGCAGTTATGGTAGGAC	790	[27]
		R: AATCCCTTTAGTTTGGGCATC		
*plaC*	ABC transporter	F: GGCGTCTTTCTTGCTTTTG	301	[21]
		R: ACCCGTTGTTCCCATAGTC		
*plaD*	Accessory protein	F: TGGACTCAAAAATGGCACAA	950	[21]
		R: GGAACCACAACTAACGAGCA		
*plS*	Prebacteriocin	F: GCCTTACCAGCGTAATGCCC	320	[20]
		R: CTGGTGATGCAATCGTTAGTTT		
16S rDNA	Positive control	PLANT1 : ATCATGATTTACATTTGAGTG	996	[62]
		LOWLAC: CGACGACCATGAACCACCTGT		

*F* forward primer, *R* reverse primer.

### Amplification and characterisation of *pln* loci

Primers were designed to analyse the upstream and downstream DNA sequences of *pln* genes (Table 4) amplified from *L. plantarum* I-UL4 genomic DNA. The PCR reaction mixture and program were as described in the experiment of “Detection of *pln* genes”, but slightly longer time of 8 min was used for each extension cycle. The DNA Walking SpeedUp™ Kit II (Seegene, Germany) was used to amplify the upstream and downstream DNA sequences of *pln* genes according to the manufacturer’s recommendations when the reference DNA sequence was not available.

**Table 4:** PCR primers that designed for the upstream and downstream DNA sequence amplification of PCR amplified *pln* genes harboured in *Lactobacillus plantarum* I-UL4

Primer designation	Primer sequence (5′–3′)
brnQ1-napA1	F: ATGCTCTTTGGGATGTTTTT
	R: ACGATGAAATAGCGGTGAGG
napA1-L	F: GTGGGCTTGAGTGGTGCTAT
	R: TACTTTACGGAGCGGCATCT
R-plnD	F: AGCAGCCCCATCACTAATC
	R: AACATCTTTGCGCTGACTATT
plnD-I	F: TGAGGACAAACAGACTGGAC
	R: AGTCTGCCTTTGAGCCTAGC
plnI-EF	F: CGTTAATGGGTGATTGAGTTG
	R: CACGGATATAGTTCAAGCCATC
plnEF-G	F: CGGTCACGCAAAACTAGAAAT
	R: TCAATCACCGCTTGTAAGAAA
plnG-H	F: TTATTGGCGGTTTTAGGTCA
	R: CGCGCACCTTCAACTAAATA
plwG1	F: CGGAATGTGGACTTTGTTGT
	R: TGCTGGCTTCCATTATTTCA
brnQ1-walk	F: CCAAGGGGGTCTTTGTAGGT
	R: CCAAAGTCGCACAAGTCAGTA
plnU-helicase	F: ATTTTGAGATGCCAGTCCTGTT
	R: TGGTCGCATACGATGTCTCC
Plw	F: CGCTTGCCAATGAACAAATA
	R: CGCCAATCGGGAATTTATCA
plwGTSP	F1: AGATGAGGCGACTAGCAGTGT
	F2: GGTGAAAATTTGAGAAAGGACAG
	F3: TGTAGCACATCGACTATCAACCA

*F* forward primer, *R* reverse primer.

### DNA sequence analysis of PCR amplified fragments

The PCR products were separated by 1% (w/v) agarose gel electrophoresis. The desired DNA fragments were excised from the agarose gel using clean scalpel and purified by using Wizard® SV Gel and PCR Clean-Up System (Promega, USA). The nucleotide sequence of the amplified fragments were analysed by ABI PRISM™ 3730 × l DNA Analyzer using BigDye® Terminator v3.0 Cycle Sequencing Kit performed by First Base Laboratories Sdn. Bhd. (Malaysia).

### DNA alignment and deduced amino acid sequence analysis

The computer software, BioEdit version 7.0.5.2 [63] was used to process and assemble nucleotide sequences, to calculate the percentage identity of DNA and deduced amino acid sequences and to perform the alignment of multiple sequences. ORF-Finder program (http://www.ncbi.nlm.nih.gov/gorf/), GeneMark [64] and Glimmer [65] were then used to determine ORF. Similarity search of nucleotide sequence was performed using Basic Local Alignment Search Tool (BlastN) program (http://blast.ncbi.nlm.nih.gov/). DNA sequence located at the upstream of start codon of each ORF was searched for the putative ribosomal binding site (RBS) manually by comparing the reported DNA sequence of RBS (5′-AGGAGG-3, which is complementary to 3′ end of 16S rRNA 5′CCUCCU-3′) of *L. plantarum* [66]. Putative promoter was also searched manually by comparing amplified DNA sequence with promoter sequences reported for *pln* operons [28]. Isoelectric point (pI) and molecular mass (MW) of the deduced peptide were calculated using ExPASY Compute pI/MW program (http://expasy.org/tools/pi_tool.html) and conserved protein domains were identified using CD-search program (http://www.ncbi.nlm.nih.gov/Structure/cdd/wrpsb.cgi) available at NCBI website.

### Determination of genetic location of *pln* loci

Southern hybridisation was carried out to determine the genetic location of *pln* loci that harboured *plnEF* and *plw* structural genes were either chromosomally or plasmid encoded. Genomic DNA of *L. plantarum* I-UL4 was separated by 0.7% (w/v) agarose gel electrophoresis and visualized by UV transillumination. The separated genomic DNA bands were then depurinated, denatured and transferred onto the Immobilon-Ny+ Transfer Membrane (Milipore, USA) according to the instructions of manufacturer. Three DNA probes of 16S_probe_, EF_probe_ and W_probe_ that developed from PCR products generated from primers listed in Table 1 were labelled using the NEBlot® Phototope® Kit (New England Biolabs, USA) according to the manufacturer’s instruction. The probes were 100% specific to 16S rDNA [62], *plnEF* [22] structural gene and *plw* [19] structural gene of *L. plantarum* I-UL4, respectively. The Southern blot membrane containing separated genomic DNA bands of *L. plantarum* I-UL4 was prehybridised with DNA probes at 58°C for 40 min, followed by further hybridisation at 53°C for 18 h. The hybridised membrane was then processed and visualised further using Phototope®–Star Detection Kit (New England Biolabs, USA) performed according to the manufacturer’s instruction.

## End note

The DNA sequences for both *plw* and *plnEF* loci of *L. plantarum* I-UL4 were deposited at [GenBank/EMBL/DDBJ with accession numbers of GU322921 and GU138149] respectively.

## Abbreviations

*pln*: plantaricin
ORF: open reading frame
RBS: ribosomal binding site
IF: induction factor
HPK: histidine protein kinase
RR: response regulator
pI: isoelectric point
MW: molecular mass.

## References

[1] Ray B, Bhunia A (2008). Fundamental food microbiology.

[2] Piard JC, Desmazeud M (1991). Inhibition factors produced by lactic acid bacteria: Oxygen metabolites and catabolism end products. Le Lait.

[3] Piard JC, Desmazeud M (1992). Inhibiting factors produced by lactic acid bacteria: bacteriocins and other antibacterial substances. Le Lait.

[4] Tagg JR, Dajani AS, Wannamaker LW (1976). Bacteriocins of Gram-positive bacteria. Bacteriol Rev.

[5] Sablon E, Contreras B, Vandamme E (2000). Antimicrobial peptides of lactic acid bacteria: mode of action, genetics and biosynthesis. Adv Biochem Eng Biotechnol.

[6] Hechard Y, Sahl HG (2002). Mode of action of modified and unmodified bacteriocins from Gram-positive bacteria. Biochemie.

[7] Bauer R, Dicks LMT (2005). Mode of action of lipid II-targeting lantibiotics. Int J Food Microbiol.

[8] Hoover DG, Chen H, Davidson PM, Sofos JN, Branen AL (2005). Bacteriocins with potential for use in foods. Antimicrobials in food.

[9] Gillor O, Nigro LM, Riley MA (2005). Genetically engineered bacteriocins and their potential as the next generation of antimicrobials. Curr Pharm Des.

[10] Gillor O, Etzion A, Riley MA (2008). The dual role of bacteriocins as anti- and probiotics. Appl Microbiol Biotechnol.

[11] Galvez A, Abriouel H, Lopez RL, Omar NB (2007). Bacteriocin-based strategies for food biopreservation. Int J Food Microbiol.

[12] Dimov S, Ivanova P, Harizanova N (2005). Genetics of bacteriocins biosynthesis by lactic acid bacteria. Biotechnol Biotechnol Equip.

[13] Klaenhammer TR (1993). Genetics of bacteriocins produced by lactic acid bacteria. FEMS Microbiol Rev.

[14] Smaoui S, Elleuch L, Bejar W, Karray-Rebai I, Ayadi I, Jaouadi F (2010). Inhibition of fungi and Gram-negative bacteria by bacteriocin BacTN635 produced by *Lactobacillus plantarum* sp. TN635. Appl Biochem Biotechnol.

[15] Todorov SD, Nyati H, Meincken M, Dicks LMT (2007). Partial characterisation of bacteriocin AMA-K, produced by *Lactobacillus plantarum* AMA-K isolated from naturally fermented milk from Zimbabwe. Food Control.

[16] Todorov SD, Dicks LMT (2005). *Lactobacillus plantarum* isolated from molasses produces bacteriocins active against Gram-negative bacteria. Enzyme Microbial Technol.

[17] Todorov SD, Dicks LMT (2004). Influence of growth conditions on the production of a bacteriocin by *Lactococcus lactis* subsp. *lactis* ST34BR, a strain isolated from barley beer. J Basic Microbiol.

[18] Messi P, Bondi M, Sabia C, Battini R, Manicardi G (2001). Detection and preliminary characterisation of a bacteriocin (plantaricin 35d) produced by a *Lactobacillus plantarum* strain. Int J Food Microbiol.

[19] Holo H, Jeknic Z, Daeschel M, Stevanovic S, Nes IF (2001). Plantaricin W from *Lactobacillus plantarum* belongs to a new family of two-peptide lantibiotics. Microbiology.

[20] Stephens SK, Floriano B, Cathcart DP, Bayley SA, Witt VF, Jiménez-Díaz R (1998). Molecular analysis of the locus responsible for production of plantaricin S, a two-peptide bacteriocin produced by *Lactobacillus plantarum*. Appl Environ Microbiol.

[21] van Reenen CA, Chikindas ML, Van Zyl WH, Dicks LMT (2003). Characterisation and heterologous expression of a class IIa bacteriocin, plantaricin 423 from *Lactobacillus plantarum* 423, in *Saccharomyces cerevisiae*. Int J Food Microbiol.

[22] Diep DB, Havarstein LS, Nes IF (1996). Characterisation of the locus responsible for the bacteriocin production in *Lactobacillus plantarum* C11. J Bacteriol.

[23] Kleerebezem M, Boekhorst J, van Kranenburg R, Molenaar D, Kuipers OP, Leer R (2003). Complete genome sequence of *Lactobacillus plantarum* WCFS1. Proc Natl Acad Sci.

[24] Zhang ZY, Liu C, Zhu YZ, Zhong Y, Zhu YQ, Zheng HJ (2009). Complete genome sequence of *Lactobacillus plantarum* JDM1. Genome Announc.

[25] Rojo-Bezares B, Sáenz Y, Navarro L, Jiménez-Díaz R, Zarazaga M, Ruiz-Larrea F (2008). Characterisation of a new organisation of the plantaricin locus in the inducible bacteriocin-producing *Lactobacillus plantarum* J23 of grape must origin. Archiv Microbiol.

[26] Navarro L, Rojo-Bezares B, Saenz Y, Diez L, Zarazaga M, Ruiz-Larrea F (2008). Comparative study of the *pln* locus of the quorum-sensing regulated bacteriocin-producing *L. Plantarum* J51 strain. Int J Food Microbiol.

[27] Maldonado A, Ruiz-Barba JL, Jimenez-Diaz R (2003). Purification and genetic characterisation of plantaricin NC8, a novel coculture-inducible two-peptide bacteriocin from *Lactobacillus plantarum* NC8. Appl Environ Microbiol.

[28] Diep DB, Straume D, Kjos M, Torres C, Nes IF (2009). An overview of the mosaic bacteriocin *pln* loci from *Lactobacillus plantarum*. Peptides.

[29] Loh TC, Thanh NT, Foo HL, Bejo MH, Azhar BK (2010). Feeding of different levels of metabolite combinations produced by *Lactobacillus plantarum* on growth performance, fecal lactic acid bacteria and Enterobacteriaceae count, volatile fatty acids and villi height in broilers. J Anim Sci.

[30] Loh TC, Chong SW, Foo HL, Law FL (2009). Effects on growth performance, faecal microflora and plasma cholesterol after supplementation of spray-dried metabolite to postweaning rats. Czech J Animal Sci.

[31] Loh TC, Harun HA, Foo HL, Law FL (2008). Effects of feeding spray-dried metabolites of *Lactococcus lactis* subsp. *lactis*–RW18 in post-weaning rats. Int J Probiotics Prebiotics.

[32] Loh TC, Lee TM, Foo HL, Law FL, Rajion MA (2008). Growth performance and fecal microflora of rats offered metabolites from lactic acid bacteria. J Appl Animal Res.

[33] Thanh NT, Loh TC, Foo HL, Bejo MH, Azhar BK (2009). Effects of feeding metabolite combinations produced by *Lactobacillus**plantarum* on growth performance, faecal microbial population, small intestine villus height and faecal volatile fatty acids in broilers. Br Poult Sci.

[34] Foo HL, Loh TC, Law FL, Lim YS, Kufli CN, Rusul G (2003). Effect of feeding *Lactobacillus plantarum* I-UL4 isolated from Malaysian Tempeh on growth performance, faecal flora and lactic acid bacteria and plasma cholesterol concentrations in postweaning rats. Food Sci Biotechnol.

[35] Lim YS (2003). Master thesis.

[36] Thanh NT, Loh TC, Foo HL, Bejo MH, Kasim AB (2010). Inhibitory activity of metabolites produced by strains of *Lactobacillus plantarum* isolated from Malaysian fermented food. Int J Probiotics Prebiotics.

[37] Moghadam MS, Foo HL, Leow TC, Raha AR, Loh TC (2010). Novel bacteriocinogenic *Lactobacillus plantarum* strains and their differentiation by sequence analysis of 16S rDNA, 16S-23S and 23S-5S intergenic spacer regions and randomly amplified polymorphic DNA analysis. Food Technol Biotechnol.

[38] Omar NB, Abriouel H, Lucas R, Martinez-Canamero M, Guyot JP, Galvez A (2006). Isolation of Bacteriocinogenic *Lactobacillus plantarum* strains from ben saalga, a traditional fermented gruel from Burkina Faso. Int J Food Microbiol.

[39] Omar NB, Abriouel H, Keleke S, Sanchez Valenzuela A, Martinez-Canamero M, Lucas Lopez R (2008). Bacteriocin-producing Lactobacillus strains isolated from poto poto, a Congolese fermented maize product, and genetic fingerprinting of their plantaricin operons. Int J Food Microbiol.

[40] Knoll C, Divol B, du Toit M (2008). Genetic screening of lactic acid bacteria of oenological origin for bacteriocin encoding genes. Food Microbiol.

[41] Risoen PA, Havarstein LS, Diep DB, Nes IF (1998). Identification of the DNA-binding sites for two response regulators involved in control of bacteriocin synthesis in *Lactobacillus plantarum* C11. Mol Gen Genet.

[42] Risoen PA, Brurberg MB, Eijsink VGH, Nes IF (2000). Functional analysis of promoters involved in quorum sensing-based regulation of bacteriocin production in *Lactobacillus*. Mol Microbiol.

[43] Risoen PA, Johnsborg O, Diep DB, Hamoen L, Venema G, Nes IF (2001). Regulation of bacteriocin production in *Lactobacillus**plantarum* depends on a conserved promoter arrangement with consensus binding sequence. Mol Genet Genomics.

[44] Axelsson L, Holck A (1995). The genes involved in production of and immunity to Sakacin A, a bacteriocin from *Lactobacillus sake* Lb706. J Bacteriol.

[45] Diep DB, Axelsson L, Grefsli C, Nes IF (2000). The synthesis of the bacteriocin sakacin A is a temperature-sensitive process regulated by a pheromone peptide through a three-component regulatory system. Mircobiology.

[46] Huhne K, Axelsson L, Holck A, Krockel L (1996). Analysis of the sakacin P gene cluster from *Lactobacillus sake* LB674 and its expression in sakacin-negative *Lb. sake* strains. Microbiology.

[47] Eijsink VGH, Brurberg MB, Middelhoven PH, Nes IF (1996). Induction of bacteriocin production in *Lactobacillus sake* by a secreted peptide. J Bacteriol.

[48] Franz CMAP, van Belkum MJ, Worobo RW, Vederas JC, Stiles ME (2000). Characterisation of the genetic locus responsible for production and immunity carnobacteriocin A: the immunity gene confers cross-protection to enterocin B. Microbiology.

[49] Quadri LEN, Kleerebezem M, Kuipers OP, de Vos WM, Stiles ME (1997). Characterisation of a locus from *Carnobacterium piscicola* LV17B involved in bacteriocin production and immunity: evidence for global inducer-mediated transcriptional regulation. J Bacteriol.

[50] Nilsen T, Nes IF, Holo H (1998). An exported Inducer peptide regulates bacteriocin production in *Enterococcus faecium* CTC492. J Bacteriol.

[51] O’Keeffe T, Hill C, Ross RP (1999). Characterisation and heterologous expression of the genes encoding enterocin A production, immunity, and regulation in *Enterococcus faecium* DPC1146. Appl Environ Microbiol.

[52] Diep DB, Havarstein LS, Nes IF (1995). A bacteriocin-like peptide induces bacteriocin synthesis in *Lactobacillus plantarum* C11. Mol Microbiol.

[53] Kleerebezem M, Kuipers OP, de Vos WM, Stiles ME, Quadri LEN (2001). A two-component signal-transduction cascade in *Carnobacterium piscicola* LV17B: two signaling peptides and one sensor-transmitter. Peptides.

[54] Havarstein LS, Holo H, Nes IF (1994). The leader peptide of colicin V shares consensus sequences with leader peptides that are common among peptide bacteriocins produced by Gram-positive bacteria. Microbiology.

[55] Pei J, Grishin NV (2001). Type II CAAX prenyl endopeptidases belong to a novel superfamily of putative membrane-bound metalloproteases. Trends Biochem Sci.

[56] Kjos M, Snipen L, Salehian Z, Nes IF, Diep DB (2010). The Abi proteins and their involvement in bacteriocin self-immunity. J Bacteriol.

[57] Datta V, Myskowski SM, Kwinn LA, Chiem DN, Varki N, Kansal RG (2005). Mutational analysis of the group A streptococcal operon encoding streptolysin S and its virulence role in invasive infection. Mol Microbiol.

[58] Lux T, Nuhn M, Hakenbeck R, Reichmann P (2007). Diversity of bacteriocins and activity spectrum in *Streptococcus pneumonia*. J Bacteriol.

[59] de Man JC, Rogasa M, Sharpe ME (1960). A medium for the cultivation of *lactobacilli*. J Appl Bacteriol.

[60] de los Reyes-Gavilán CG, Limsowtin GK, Tailliez P, Sechaud L, Accolas JP (1992). *Lactobacillus helveticus*-specific DNA probe detects restriction fragment length polymorphisms in this species. Appl Environ Microbiol.

[61] Rozen S, Skaletsky HJ (2000). Primer3 on the WWW for general users and for biologist programmers. Methods Mol Biol.

[62] Chagnaud P, Machinis K, Coutte LA, Marecat A, Mercenier A (2001). Rapid PCR-based procedure to identify lactic acid bacteria: application to six common *Lactobacillus* species. J Microbiol Methods.

[63] Hall TA (1999). BioEdit: a user-friendly biological sequence alignment editor and analysis program for Windows 95/98/NT. Nucleic Acids Symp Ser.

[64] Lukashin A, Borodovsky M (1998). GeneMark.hmm: new solutions for gene finding. Nucleic Acids Res.

[65] Delcher AL, Bratke KA, Powers EC, Salzberg SL (2007). Identifying bacterial genes and endosymbiont DNA with Glimmer. Bioinformatics.

[66] Pouwels PH, Leer RJ (1993). Genetics of *lactobacilli*: plasmids and gene expression. Antonie Van Leeuwenhoek.

